# An Incidental Finding of Butterfly Vertebrae in a Case of Vertebral Defects, Anal Atresia, Cardiac Defects, Tracheo-Esophageal Fistula, Renal Anomalies, and Limb Abnormalities (VACTERL)

**DOI:** 10.7759/cureus.33401

**Published:** 2023-01-05

**Authors:** Ashritha Rao, Sarika Gaikwad, Amar Taksande, Mayur B Wanjari

**Affiliations:** 1 Department of Pediatrics, Jawaharlal Nehru Medical College, Datta Meghe Institute of Medical Sciences, Wardha, IND; 2 Research Scientist, Jawaharlal Nehru Medical College, Datta Meghe Institute of Medical Sciences, Wardha, IND

**Keywords:** vacterl, lower back pain (lbp), tetralogy of fallot., anal atresia, butterfly vertebrae

## Abstract

Butterfly spine is a rare benign congenital abnormality. The onset of a minimum of three of the congenital malformations of vertebral defects, anal atresia, cardiac defects, tracheo-esophageal fistula, renal anomalies, and limb abnormalities often characterises the VATER/VACTERL relationship. Recognising this anomaly is crucial for diagnosis, although this rare aberration is thought to be asymptomatic most of the time. Here we are describing a case of a one-year-old female child who has tetralogy of Fallot, congenital anal atresia, vesicovaginal fistula, and butterfly vertebrae which were found as an incidental finding. Furthermore we suggest screening all the children with any one abnormality of VACTERL, usually vertebral anomalies are screened.

## Introduction

The butterfly vertebra is a rare and often benign congenital spine abnormality of the vertebrae. It might be associated with kyphoscoliosis [1]. The butterfly vertebra, also labeled as the cleft vertebra, sagittal cleft vertebra, anterior rachischisis, and anterior spina bifida, was originally identified in 1844 [2,3]. It frequently affects the lumbar spine and can develop alone or in conjunction with other systemic deformities of the renal, digestive, skeletal, and nervous systems. 

It’s a sagittal deformity in the vertebral body as the two lateral chondrification centers fail to fuse together during development. The name is based on the pattern that the two hemivertebrae appear on x-rays as butterfly wings from the central gap [1]. According to the research, this deformity is highly unusual. The link between butterfly vertebrae and congenital illnesses such as Alagille syndrome, Jarcho-Levin syndrome, Crouzon syndrome, and Pfeiffer's condition has been reported in a number of sources [3]. Butterfly vertebrae have also been associated with reports of low back pain. They are uncommon. Thus it is simple to mistake them for other disease processes or traumatic compression fractures. Since many are only discovered by chance, the true prevalence and the complete range of connected disorders are unknown [1].

## Case presentation

We present a case of a one-year-old female child brought by her parents to the tertiary care hospital with complaints of an imperforate anus since birth and a history of the passage of stools from the vagina. She had a history of nonpassage of stools from two days; later, she passed stools all at once. Her history of abdominal distension is why the child was brought to the hospital for further management.

**Figure 1:**
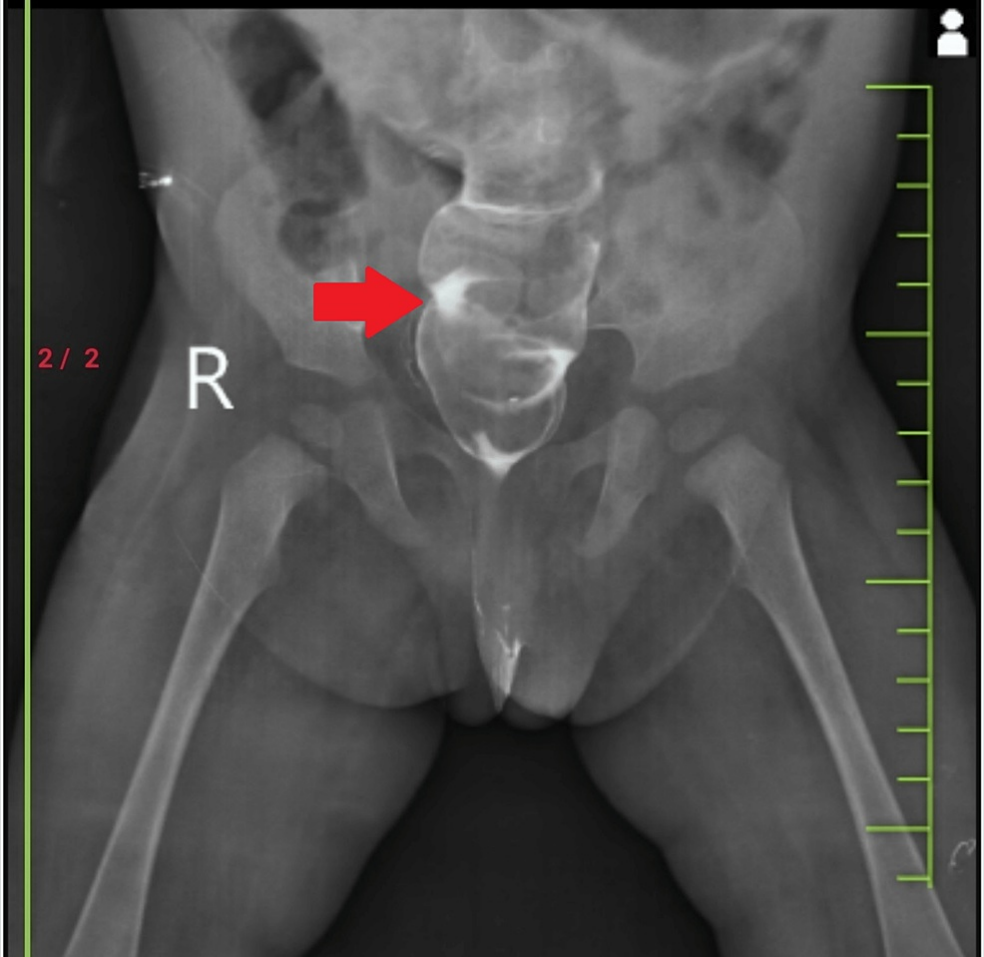
X-Ray lumbosacral spine anteroposterior view showing widening of the L2/L3,L4 vertebrae

**Figure 2:**
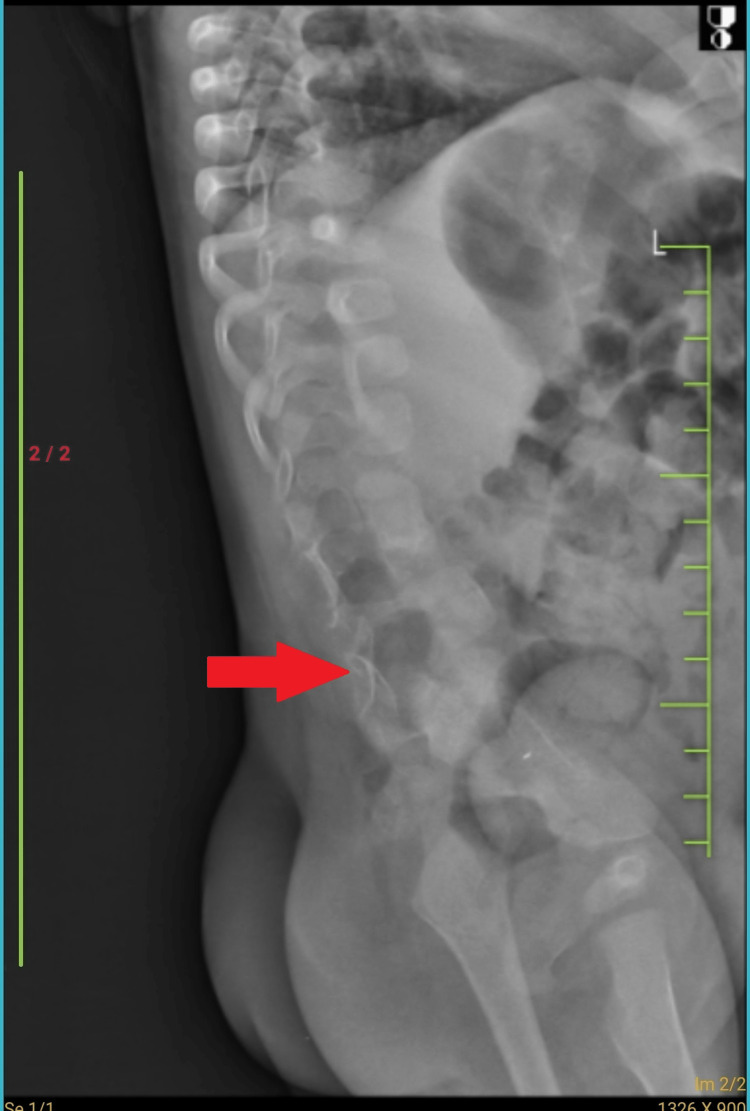
X-Ray lumbosacral spine lateral view showing altered intervertebral discs showing concavities along the adjacent end plates.

The child had a gross motor developmental delay; clinically, the child had mild lumbar kyphoscoliosis, but no lower back tenderness, and local examinations were suggestive of fused vertebrae. X-ray lumbosacral (LS) spine (anteroposterior [AP] and lateral view) was done, showing widening of the L2/L3 and L4 vertebrae and altered intervertebral discs showing concavities along the adjacent end plates suggestive of butterfly vertebrae (Figure 1, 2).

**Figure 3:**
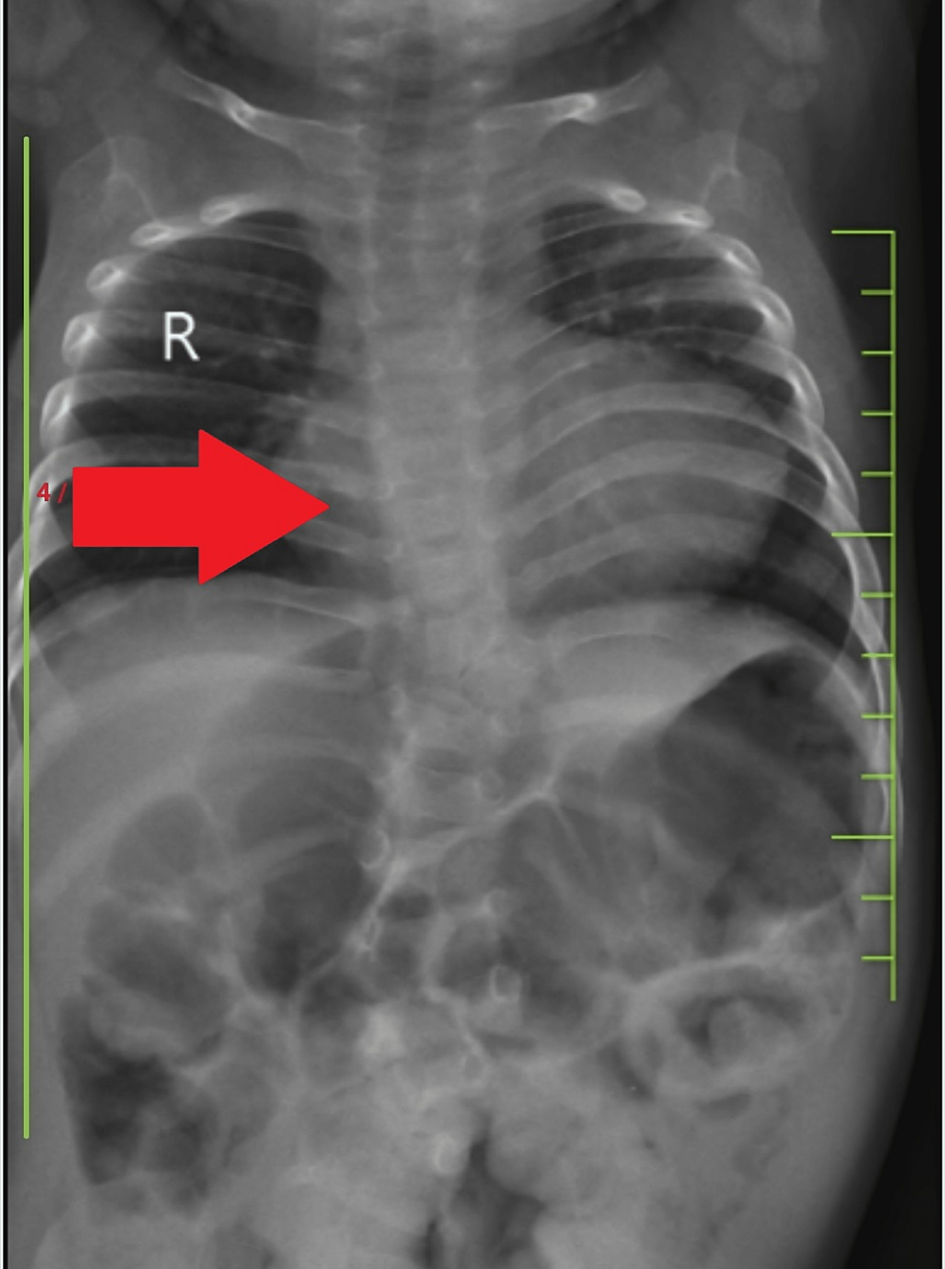
X-Ray Chest posteroanterior view suggesting boot-shaped heart

The child is also a known case of congenital heart disease (tetralogy of Fallot; TOF) and presented with no cyanosis clinically; on auscultation, there was an ejection systolic murmur best appreciated along the left mid sternal border on X-Ray posteroanterior (PA) view it showed a boot-shaped heart suggestive of TOF (Figure 3). The child had no episodes of cyanotic spells and was treated medically with propranolol; they were advised for complete surgical repair with ventricular septal defect (VSD) closure and removal of the pulmonic stenosis on follow-up. There was no history of similar complaints in the family. The child was taken for surgery for a rectovaginal fistula performed. Anterior sagittal anorectoplasty with a high sigmoid colostomy was done. Postoperatively child was managed conservatively. Eventually, the child was stable. For the kyphoscoliosis, the child was started with physiotherapy and was advised to have proper posturing and was discharged and asked for follow-up after three months.

## Discussion

In between the third and sixth weeks of pregnancy, during somitogenesis, the butterfly vertebra develops in utero [3,4]. The notochord is "compressed out" into the intervertebral disc, which results in the genesis of the nucleus pulposus [4]. The butterfly vertebral malformation occurs from the non-union of both sides of the vertebral body, which is thought to be caused by lingering remains of the notochord [5,6].

Usually, the vertebrae and intervertebral discs above and below elongate toward the midline to make up for the deficiency. The defect is frequently discovered by accident radiologically, but in our instance, we made a clinical diagnosis and radiological confirmation of it, even though it might be connected to lower back pain. This abnormality may be connected to various spinal abnormalities such as spina bifida, diastematomyelia, lumbosacral transitional vertebrae, Alagille and Klippel-Feil syndrome, as well as digestive and renal abnormalities. The etiology is thought to be genetic (chromosome 20 deletions) or congenitally vascularization-deficient. Butterfly vertebra typically has no symptoms. Patients with this deformity may present with unusual chronic lower back pain [7,8] or may have an inadvertent diagnosis, as in our case. The butterfly vertebra looks wedge-shaped in lateral view on plain radiography, which is usually mistaken for a compression fracture [3,5].

A dual-energy X-ray absorptiometry (DEXA) test can be done to differentiate neoplasia, osteoporosis, or infection. There have been occurrences of butterfly vertebrae linked to disc protrusion or nucleus pulposus herniation at the level of the aberrant vertebral body. Additional diagnostic techniques are not required once a diagnosis has been made. This congenital defect, usually mistaken as a spinal fracture, must be known to neurosurgeons. As per our knowledge, this is the second case reported in the pediatric age group [6].

## Conclusions

Early diagnosis of the butterfly vertebrae is necessary to initiate early treatment modalities such as physiotherapy and postural corrections to prevent morbidity in adulthood. Even though it is mostly an asymptomatic condition, early screening in the pediatric age group is always a better idea to prevent further complications and is advised.
